# Simplified detection of polyhistidine-tagged proteins in gels and membranes using a UV-excitable dye and a multiple chelator head pair

**DOI:** 10.1074/jbc.RA120.014132

**Published:** 2020-07-09

**Authors:** Vlad-Stefan Raducanu, Ioannis Isaioglou, Daniela-Violeta Raducanu, Jasmeen S. Merzaban, Samir M. Hamdan

**Affiliations:** Division of Biological and Environmental Sciences and Engineering, King Abdullah University of Science and Technology, Thuwal, Saudi Arabia

**Keywords:** physical method, Western blot, fluorescence, imaging, recombinant protein expression, metal-ion-chelating nitrilotriacetate (NTA), His-tag detection, blot membrane, UV detection of His-tag protein, PAGE, UV transilluminator, UVHis-PAGE, histidine

## Abstract

The polyhistidine tag (His-tag) is one of the most popular protein tags used in the life sciences. Traditionally, the detection of His-tagged proteins relies on immunoblotting with anti-His antibodies. This approach is laborious for certain applications, such as protein purification, where time and simplicity are critical. The His-tag can also be directly detected by metal ion–loaded nickel-nitrilotriacetic acid–based chelator heads conjugated to fluorophores, which is a convenient alternative method to immunoblotting. Typically, such chelator heads are conjugated to either green or red fluorophores, the detection of which requires specialized excitation sources and detection systems. Here, we demonstrate that post-run staining is ideal for His-tag detection by metal ion–loaded and fluorescently labeled chelator heads in PAGE and blot membranes. Additionally, by comparing the performances of different chelator heads, we show how differences in microscopic affinity constants translate to macroscopic differences in the detection limits in environments with limited diffusion, such as PAGE. On the basis of these results, we devise a simple approach, called UVHis-PAGE, that uses metal ion–loaded and fluorescently labeled chelator heads to detect His-tagged proteins in PAGE and blot membranes. Our method uses a UV transilluminator as an excitation source, and the results can be visually inspected by the naked eye.

The polyhistidine tag (His-tag) is widely used for protein purification, detection, and immobilization (1). The majority of expression vectors use a His-tag composed of six consecutive histidine residues (His_6_-tag), and this tag is fused to the protein of interest with or without flexible linkers (2). In general, fast and selective detection of His-tagged proteins is desired. With these considerations in mind, here we build a detection system for His-tagged proteins based on simple, commercially available, and cost-effective consumables and instrumentation. This system, which we call UVHis-PAGE, allows for the detection of His_6_-tagged proteins in PAGE and blot membranes using a simple UV transilluminator (Fig. 1).

**Figure 1.**
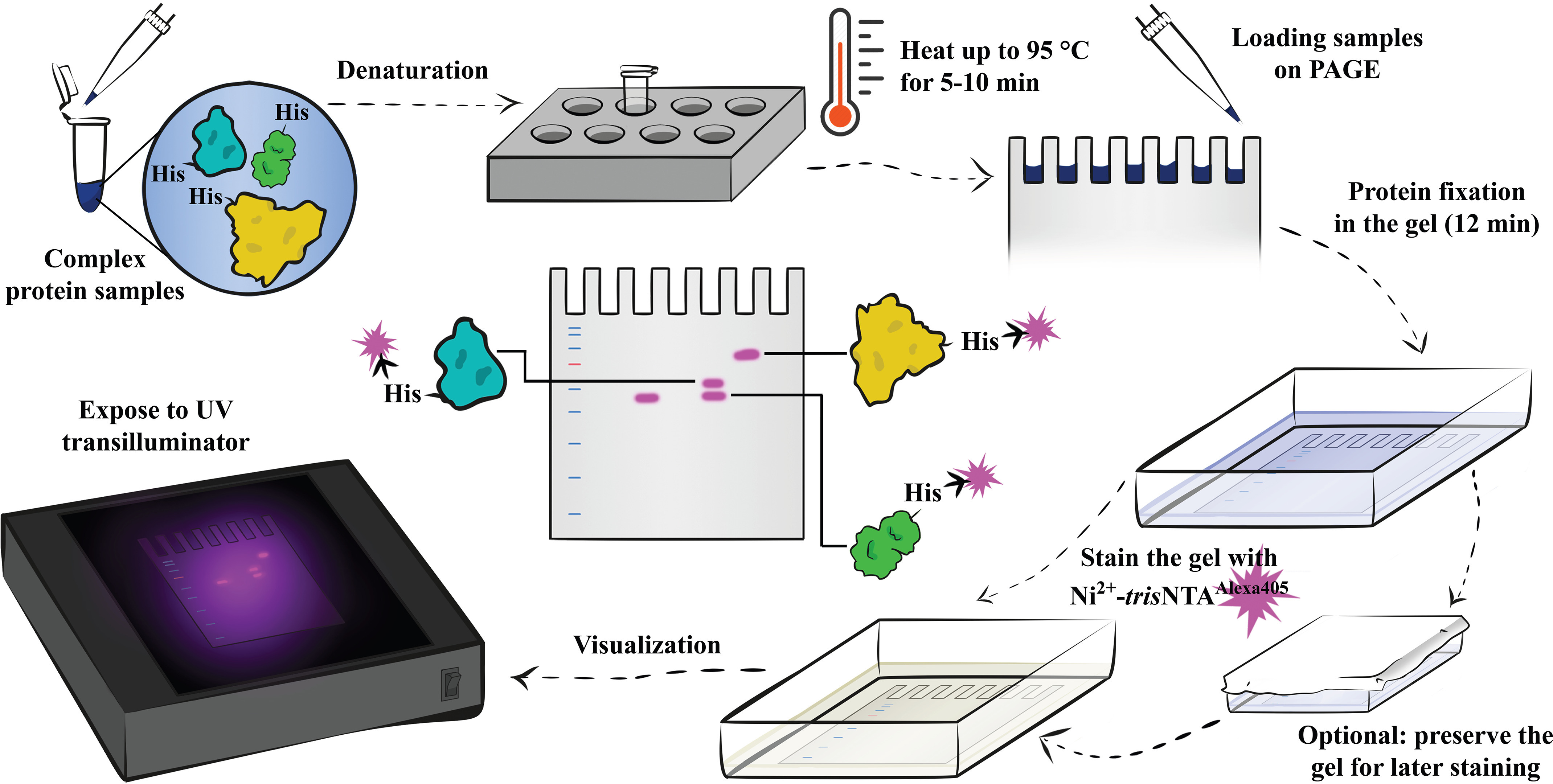
**Schematic representation of the UVHis-PAGE protocol for UV-based detection of His-tagged proteins in PAGE gels without the need for immunoblotting.** The method relies on the metal affinity interaction of the metal ion–loaded and fluorescently labeled MCH with the His-tag of the protein of interest. The conjugated fluorophore is UV-excitable with visible emission for visualization by the naked eye upon exposure to a UV transilluminator. The gel shown on the UV transilluminator is an example for illustration purposes.

As an alternative to traditional immunodetection with anti-His antibodies, metal ion–loaded nitrilotriacetate (NTA) moiety (*mono*NTA, monovalent nickel-nitriloacetic acid) (Fig. 2*a*) offers easier detection while eliminating the need for costly antibodies (3, 4). This method relies on the metal affinity interaction of the metal ion–loaded chelator head with the His-tag in an analogous manner to the method used to purify His-tagged proteins by immobilized metal affinity chromatography (2) (see also 18–22). However, the relatively weak affinity of Ni^2+^-*mono*NTA (Nickel(II)-loaded *mono*NTA) toward the commonly used His_6_-tag, in the low-micromolar range, limits the detection sensitivity. Careful chemical and geometrical considerations have led to the assembly of the NTA moiety into higher-order structures, known as multivalent chelator heads (MCHs). The multivalent interactions of Ni^2+^-MCHs (Nickel(II)-loaded MCH) increase the affinity toward His_6_-tag by a 1000-fold relative to Ni^2+^-*mono*NTA (5–9). This optimization considerably increases both the applicability and popularity of His–Ni^2+^-NTA–based approaches (10–15). Among MCHs, the commercially available *tris*NTA (trivalent nickel-nitriloacetic acid) represents the minimal lock-and-key chelator with a dissociation constant of ∼10 nm for the His_6_-tag (in its cyclic version) (Fig. 2*a*) (8). More recently, two *tris*NTA heads were coupled via an optimized linker into a super-chelator (*hexa*NTA, hexavalent nickel-nitriloacetic acid). This super-chelator further enhances the affinity toward His-tags down to the picomolar range. However, this enhancement requires an increase in the size of the His-tag to 12 consecutive histidine residues (His_12_) (16).

**Figure 2.**
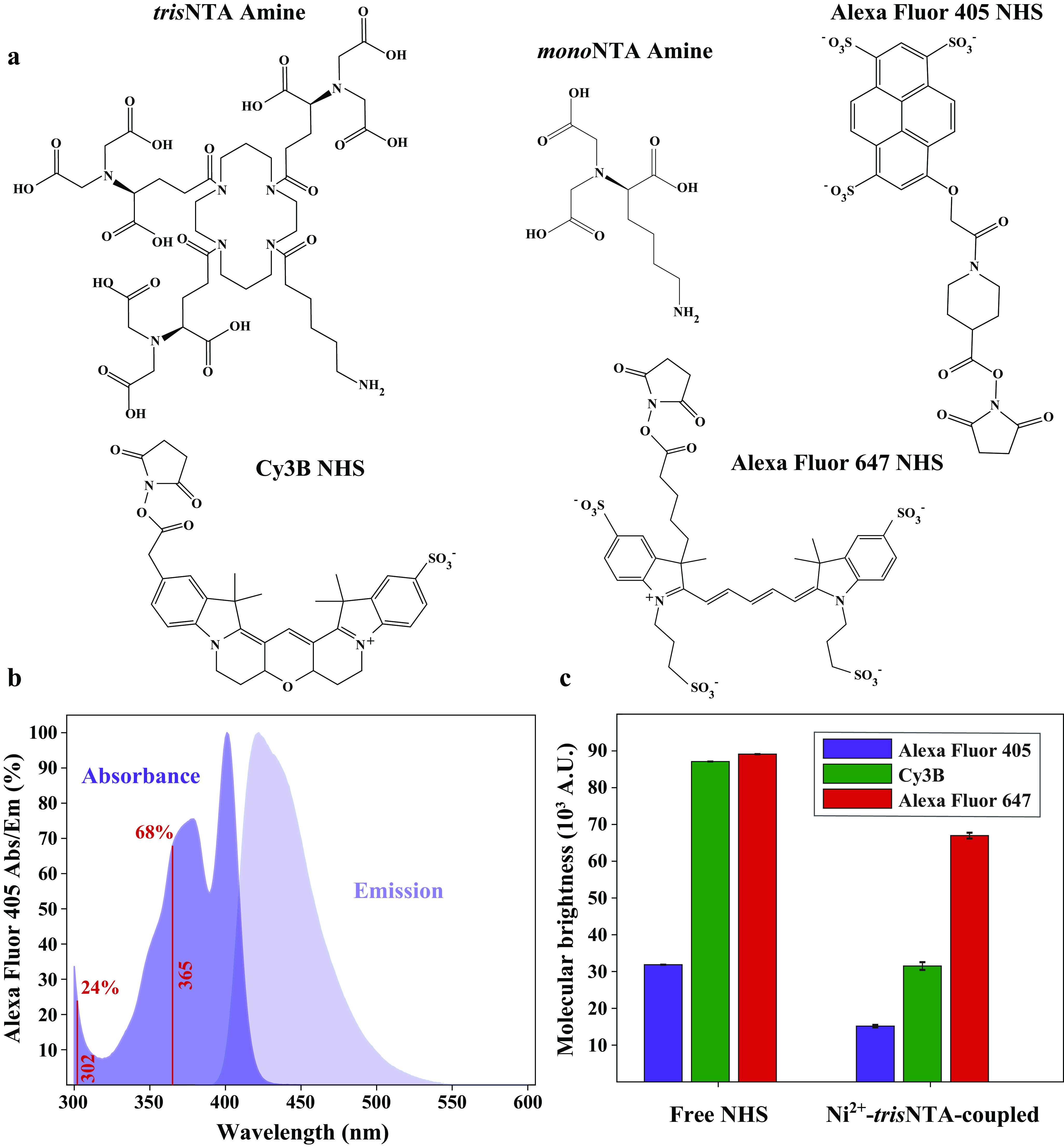
**Structure and properties of the various chemical reagents.** *a*, chemical structures of cyclic *tris*NTA containing a terminus amine group, *mono*NTA containing a terminus amine group, Alexa Fluor 405 NHS ester, Cy3B NHS ester, and Alexa Fluor 647 NHS ester. *b*, absorption and emission spectra of Alexa Fluor 405 NHS ester as available from the online Spectra Viewer tool from the Chroma Technology Corporation website. The *red vertical bars* represent the relative absorbance (%) of Alexa Fluor 405 at the indicated wavelength as compared with the maximum of absorbance. *c*, bar chart of the brightness of Alexa Fluor 405, Cy3B, and Alexa Fluor 647 as free NHS esters (*left set of bars*) and Ni^2+^-*tris*NTA conjugates (*right set of bars*). Excitation is considered to be performed at the wavelength of maximum excitation as indicated in Table S1. Each brightness was estimated as described in the supporting information “Experimental procedures” section and Table S1. The color code is described in the *inset legend*.

One area of applicability of MCHs, namely the detection of His-tagged proteins in PAGE has been partially overlooked until the recent introduction of the HisQuick-PAGE assay (17). In this assay, the authors relied on the preincubation of His-tagged proteins with Ni^2+^-MCH conjugate Ni^2+^-*tris*NTA^Alexa647^ or Ni^2+^–super-chelator conjugate Ni^2+^-*hexa*NTA^Alexa647^, followed by in-gel detection of the complex (pre-run staining conditions). Under denaturing conditions, Ni^2+^-*hexa*NTA^Alexa647^ could detect as low as 0.2 pmol of a His_12_-tagged protein, which is comparable to the detection limit of immunoblotting (17). In contrast, Ni^2+^-*tris*NTA^Alexa647^ failed to detect the His_12_-tagged protein under the same conditions even when used at concentrations as high as hundreds of nm; many folds above its dissociation constant (17). This apparent discrepancy between the high binding affinity of Ni^2+^-*tris*NTA and its lack of detection (17) poses several limitations to the HisQuick-PAGE assay, especially in the case of SDS-PAGE detection. From a practical perspective, His-tag detection that is based on Ni^2+^-*hexa*NTA conjugates is limited to the detection of the less-common His_12_ and with reduced detection efficiency of His_10_. From a theoretical perspective, the complete lack of detection with Ni^2+^-*tris*NTA conjugate, despite its relatively high binding strength, suggests that pre-run staining conditions may not be optimal for achieving the full detection limit of metal ion–loaded MCH conjugates. Additionally, without the possibility to compare with another chelator head, it is unclear whether the 0.2 pmol detection limit is imposed by the chelator head itself or by the conjugated fluorophore.

In the current study, we switch to post-run staining conditions (Fig. 1) to bypass the aforementioned limitations of pre-run staining conditions. We show that under post-run staining conditions, Ni^2+^-*tris*NTA green and red fluorescent conjugates can detect as low as 0.1 pmol of His_6_-tag protein. This detection limit is comparable to the pre-run staining conditions in the HisQuick-PAGE assay described in Bruchert *et al.* (17) but is achieved using the simpler *tris*NTA MCH and the widely used His_6_-tag. Moreover, we establish that detection by Ni^2+^-*tris*NTA conjugates is considerably superior to detection by Ni^2+^-*mono*NTA conjugates, even under in-gel diffusion conditions. From the fluorophore point of view, most chelator heads to date have been conjugated either to green or red fluorescent organic dyes (such as Cy3B and Alexa Fluor 647) (Fig. 2*a*), which require complex and rather costly instrumentation with specialized excitation sources and detectors. To further improve the ease of use and to increase the availability of this method, we conjugate *tris*NTA to Alexa Fluor 405 (Fig. 2*a*), which is a UV-excitable dye that emits in the visible spectrum. This approach allows us to visualize His_6_-tag proteins, with a detection limit as low as 5 pmol for SDS-PAGE or 2.5 pmol in the blot membrane, simply by using a UV transilluminator as an excitation source and a bench camera or even the naked eye for visualization.

## Results

To build the UV–based detection system for His_6_-tagged proteins in PAGE and blotting membrane, two initial choices must be made: a chelator head and a UV-excitable dye. However, given the lack of sensitivity of the chelator heads to the His_6_-tag under pre-run staining conditions (17), an alternative staining procedure that allows and maximizes the detection of His_6_-tagged proteins must be optimized first.

### Choice of chelator head and staining method for highly sensitive fluorescent detection

We hypothesized that reducing the harsh conditions of SDS-PAGE during the initial incubation and complex migration in the pre-run staining protocol (17) can increase the detection performance of a given chelator head. To evaluate this hypothesis, we implemented a post-run staining protocol (Fig. 1) that enabled us to compare the detection limit of Ni^2+^-*mono*NTA- and Ni^2+^-*tris*NTA-coupled fluorophores directly. As a target protein for detection, we used a His_6_-SUMO fusion protein which bears a single His_6_-tag at the N terminus and is >95% pure (Fig. S2*a*). Therefore, any amount of protein described in moles will correspond to the same amount of His_6_. Moreover, we preferred this protein fusion because of its relatively small size, which minimizes the chance of unspecific binding.

**Figure 3.**
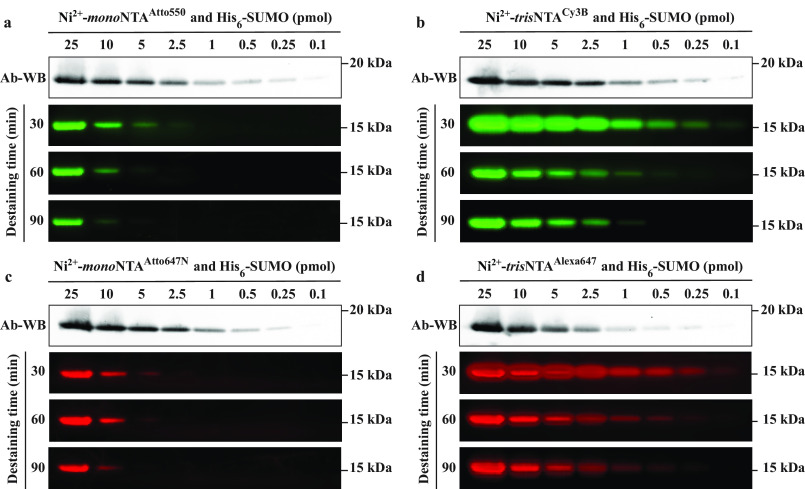
**Comparison of the detection performance of Ni^2+^-*mono*NTA and Ni^2+^-*tris*NTA fluorescent conjugates in SDS-PAGE.** *a–d*, each gel was run in duplicate and one copy was stained post-run and fixation with (*a*) Ni^2+^-*mono*NTA^Atto550^, (*b*) Ni^2+^-*tris*NTA^Cy3B^, (*c*) Ni^2+^-*mono*NTA^Atto647^, and (*d*) Ni^2+^-*tris*NTA^Alexa647^. The gels were destained for the indicated amount of time (30, 60, or 90 min) and imaged using a Typhoon laser-based scanning system (*rows 2–4 in each panel*). The second copy of each gel was used for chemiluminescent immunoblotting using an anti-His antibody (*Ab-WB*, *first row in each panel*). For all SDS-PAGE gels, the 15 kDa molecular weight marker tick corresponds to the run of the 15 kDa band of PageRuler Prestained Protein Ladder (Thermo Fisher Scientific, 26616); the ladder is run under identical conditions, stained with CBB and visualized under white light. The relative position of His_6_-SUMO protein to all bands on the PageRuler Prestained Protein Ladder can be visualized in Fig. 5*a* and Fig. S2*a*. For all Ab-WBs, the 20 kDa molecular weight marker tick corresponds to the run of the 20 kDa band of MagicMark XP Western Protein Standard (Thermo Fisher Scientific, LC5603); the ladder is run and stained under identical conditions and visualized through chemiluminescence. The relative position of His_6_-SUMO protein to all bands on the MagicMark XP Western Protein Standard can be visualized in Fig. 5*b*.

We mixed various amounts (0.1–25 pmol) of His_6_-SUMO with SDS-PAGE electrophoresis sample buffer, which we heated to 95°C and ran on 10% SDS-PAGE, as described under “Experimental procedures.” Each sample was prepared as a double loading volume (30 µl) and then split into two independent gels. This procedure was repeated four times, once for each of the tested Ni^2+^-loaded fluorophore-chelator head conjugates: Ni^2+^-*mono*NTA^Atto550^, Ni^2+^-*tris*NTA^Cy3B^, Ni^2+^-*mono*NTA^Atto647N^, and Ni^2+^-*tris*NTA^Alexa647^. One copy of each gel was transferred to PVDF membrane and immunoblotted overnight with commercial anti-His antibody and imaged with chemiluminescence (*first rows* in Fig. 3, *a*–*d*). This immunoblotting step served a 2-fold purpose. First, it ensured that equal amounts of His_6_-SUMO were loaded on each of the four replicated gels. Second, it illustrated the detection limit of typical anti-His antibody–based chemiluminescent immunoblotting as ∼0.1 pmol (*first rows* in Fig. 3, *a*–*d*).

The second copy of each of the four gels was fixed using the fast protocol (12 min) described under “Experimental procedures.” After fixing and washing the gels, the protocol proceeded directly to the staining step. Alternatively, the fixed gel can be stored in water for staining in the future with minimal diffusion of the bands (Fig. 1). For the gel staining step, fresh PBS solutions containing 150 nm Ni^2+^-*mono*NTA^Atto647N^, Ni^2+^-*tris*NTA^Alexa647^, 300 nm of Ni^2+^-*mono*NTA^Atto550^, or Ni^2+^-*tris*NTA^Cy3B^ were prepared. The gels were submerged in these solutions, and staining was allowed to proceed for 1 h at room temperature with gentle shaking in the dark.

After completion of the staining step, the gels were rinsed with water to remove the excess staining solution. In the initial trials, the gels were imaged immediately, which resulted in an excessive background, especially given the high sensitivity of the laser-based Typhoon scanner (data not shown). To remove the unbound fluorophore conjugates and reduce the background, we submerged the gels in warm water (warm water facilitates the diffusion of the unbound fluorophore conjugates out of the gels) and gently shook them in the dark for 30, 60, or 90 min. After each 30-min washing cycle, the gels were imaged using the same Typhoon imaging conditions with an appropriate excitation source and emission filters, depending on the conjugated fluorophore.

After the first destaining and imaging cycle (30 min), both Ni^2+^-*tris*NTA conjugates exhibited a His_6_-SUMO detection limit of ∼0.1 pmol (Fig. 3, *b* and *d*). In contrast, the Ni^2+^-*mono*NTA conjugates were considerably less sensitive, with a detection limit of only ∼2.5 pmol (Fig. 3, *a* and *c*). Thus, the Ni^2+^-*tris*NTA conjugates were ∼25-fold more sensitive than Ni^2+^-*mono*NTA conjugates. We further validated these detection limits by replacing His_6_-SUMO with N terminus His_6_-tagged human PCNA (its monomer has higher molecular mass (∼30 kDa) than His_6_-SUMO, and this monomer forms an oligomer (homotrimer) in solution) and staining the PCNA-containing gels with Ni^2+^-*tris*NTA^Cy3B^ or Ni^2+^- *mono*NTA^Atto550^ (Fig. S2, *b* and *c*). Each additional 30-min destaining step caused the loss of detection of the last previous band for both Ni^2+^-*tris*NTA and Ni^2+^-*mono*NTA conjugates. Nevertheless, from a quantitative point of view, this translated into the loss of only ∼0.15–0.5 pmol/30 min for Ni^2+^-*tris*NTA and ∼1–5 pmol/30 min for Ni^2+^-*mono*NTA. Therefore, in addition to the increased detection sensitivity, Ni^2+^-*tris*NTA conjugates also exhibited a ∼10-fold increase in stability over time. Overall, these results demonstrate that the post-run protocol improved the detection by Ni^2+^-*tris*NTA conjugates to a level that matches the limit of typical anti-His antibody–based chemiluminescent immunoblotting (Table S2).

### UV-excitable dye with visible emission: Trading sensitivity for detection simplicity

Equipped with the above-established highly sensitive detection provided by the post-run staining conditions, we next turned our attention to the choice of fluorophore for building a UV-based detection system for His_6_-tagged proteins. Typical UV transilluminators offer a very limited number of wavelength choices, such as 302 and 365 nm. With these considerations, by surveying the absorption spectra, molecular extinction coefficients, and quantum yields of commercially available UV-excitable fluorophores with visible emission, we chose Alexa Fluor 405. At 365 nm, this fluorophore retains ∼68% of its maximum molar extinction coefficient (Fig. 2*b*). Moreover, Alexa Fluor 405 is directly available as NHS ester for the convenience of coupling to the amine-containing chelator heads. However, Alexa Fluor 405 exhibits a relatively low brightness, which is typical for small organic UV and near-UV fluorophores (Fig. 2*c* and Table S1).

We proceeded to determine the detection limit of His_6_-tagged in SDS-PAGE and blotting membrane using Ni^2+^-*tris*NTA^Alexa405^. The samples were prepared as described in the previous section, but in the His_6_-SUMO amount range of 1–1000 pmol. Each sample was prepared as a double loading volume (30 µl) and then split into two independent gels. One copy of the gel was transferred to PVDF membrane and blocked with BSA for 1 h, and the second was fixed using the fast protocol (12 min). After rinsing, both the gel and the membrane can be either stained immediately or stored for future staining (Fig. 1).

**Figure 4.**
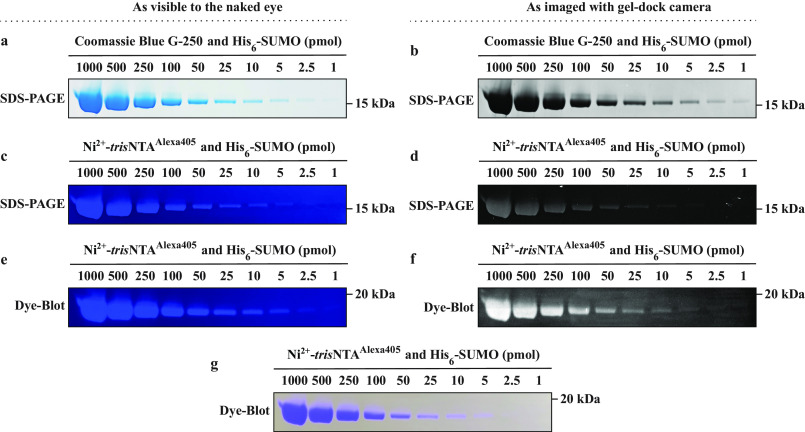
**Performance of Ni^2+^-*tris*NTA^Alexa405^ in the UVHis-PAGE protocol and comparison with CBB staining.** *a* and *b*, CBB-stained SDS-PAGE was imaged with (*a*) a regular cellphone camera and (*b*) a gel-dock camera. *c* and *d*, Ni^2+^-*tris*NTA^Alexa405^-stained SDS-PAGE was illuminated by a UV transilluminator and imaged in the dark with (*c*) a regular cellphone camera and (*d*) a gel-dock camera. *e* and *f*, an additional copy of the gel was transferred to a blotting membrane, blocked with BSA, stained with Ni^2+^-*tris*NTA^Alexa405^, illuminated by a UV transilluminator, and imaged in the dark with (*e*) a regular cellphone camera and (*f*) a gel-dock camera. *g*, Ni^2+^-*tris*NTA^Alexa405^-stained blot membrane can also be clearly visualized through the protective screen of the UV transilluminator even under ambient light conditions. For all SDS-PAGE gels, the 15 kDa molecular weight marker tick corresponds to the run of the 15 kDa band of PageRuler Prestained Protein Ladder (Thermo Fisher Scientific, 26616); the ladder is run under identical conditions, stained with CBB and visualized under white light. The relative position of His_6_-SUMO protein to all the bands on the PageRuler Prestained Protein Ladder can be visualized in Fig. 5*a* and Fig. S2*a*. For all Dye-Blots, the 20 kDa molecular weight marker tick corresponds to the run of the 20 kDa band of MagicMark XP Western Protein Standard (Thermo Fisher Scientific, LC5603); the ladder is run and stained under identical conditions as described for Fig. 5*b* and visualized through chemiluminescence. The relative position of His_6_-SUMO protein to all the bands on the MagicMark XP Western Protein Standard can be visualized in Fig. 5*b*.

For the staining step, both the gel and the membrane were treated identically. The gel and the membrane were submerged into fresh PBS solution containing 2 μm Ni^2+^-*tris*NTA^Alexa405^ and then they were submerged in this solution and incubated for 1 h in the dark with gentle shaking. After 1 h, the gel was rinsed with warm water and the membrane was rinsed with 1 × TBST and exposed to a UV transilluminator equipped with a protective screen. To capture an image as similar as possible to the one available to the naked eye, we photographed both the gel and the membrane with a regular cellphone camera in the dark (Fig. 4, *c* and *e*). For accuracy, we also took images with the integrated camera of the FluorChem Q Image analysis system (Fig. 4, *d* and *f*).

With this simple approach, a detection limit of ∼5 pmol for the gel and ∼2.5 pmol for the membrane were visible to the naked eye through the protective screen of the UV transilluminator (Fig. 4, *c* and *e*). These detection limits were also confirmed by the camera of the FluorChem Q Image analysis system (Fig. 4, *d* and *f*). Remarkably, these detection limits were achieved without the need for any additional time-consuming destaining steps, despite the relatively low brightness of Ni^2+^-*tris*NTA^Alexa405^ (Fig. 2c and Table S1) and the simplicity of the equipment involved. The membrane was easily visualized using the UV transilluminator and the naked eye, even under ambient light conditions (Fig. 4*g*). Moreover, at the end of the visualization, the gel could be stained with Coomassie Brilliant Blue (CBB) for complementarity (Fig. 4, *a* and *b*). By using the fast staining protocol described under “Experimental procedures,” CBB in its G-250 form allowed ∼1 pmol of His_6_-SUMO to be visualized. In the case of the blotting membrane, washing in the presence of at least 50 mm EDTA removed the bound Ni^2+^-*tris*NTA^Alexa405^, thus allowing the membrane to be reused for future experiments (data not shown).

To quantitatively assess the correlation between the CBB and Ni^2+^-*tris*NTA^Alexa405^ detection, we measured the band intensities in the gels and the membrane by using the built-in function of the ImageJ software. We then normalized these values to the intensity of the 1000 pmol band in the corresponding gel or membrane (Fig. S3). The intensities of Ni^2+^-*tris*NTA^Alexa405^ in both the gel and membrane scaled very accurately with the CBB intensities in the gel, with a correlation coefficient higher than 99%. From a practical point of view, this high correlation indicates excellent complementarity of Ni^2+^-*tris*NTA^Alexa405^ and CBB staining within the given detection range of 5–500 pmol.

### UVHis-PAGE is a highly specific detection system

**Figure 5.**
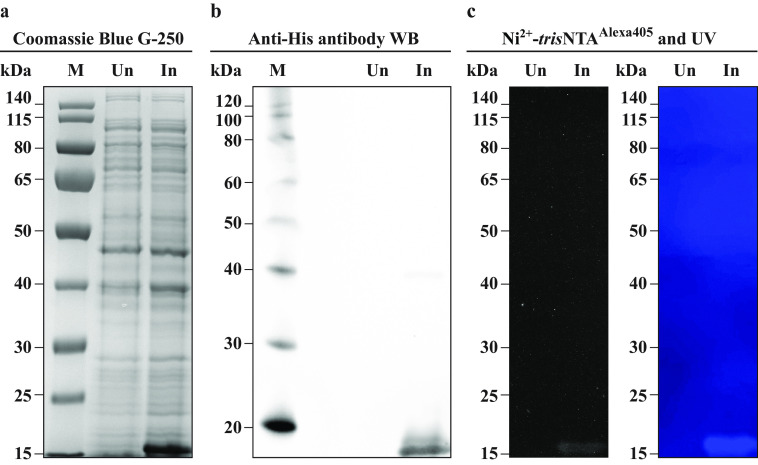
**Specificity of Ni^2+^-*tris*NTA^Alexa405^ in the UVHis-PAGE protocol and comparison with anti-His antibody-based immunoblotting.** *a*, uninduced (*Un*) and induced (*In*) samples of *E. coli* culture expressing His_6_-SUMO were run on SDS-PAGE and stained with CBB. The molecular weight marker (*M*) is PageRuler Prestained Protein Ladder (Thermo Fisher Scientific, 26616). *b*, an additional copy of this SDS-PAGE was used for chemiluminescent immunoblotting using an anti-His antibody. The molecular weight marker (*M*) is MagicMark XP Western Protein Standard (Thermo Fisher Scientific, LC5603). *c*, before CBB staining, the SDS-PAGE gel was stained with Ni^2+^-*tris*NTA^Alexa405^, illuminated by a UV transilluminator, and imaged in the dark with a regular cellphone camera (*right*) and a gel-dock camera (*left*). The molecular weight marker ticks (kDa) correspond to the run of the bands of PageRuler Prestained Protein Ladder obtained by overlapping these images under UV exposure.

In complex mixture samples, especially in whole cellular extracts, a variety of different proteins can show different affinities toward His-tag–binding reagents, such as anti-His antibodies and metal ion–loaded chelator heads (23–27). Therefore, we investigated the specificity of Ni^2+^-*tris*NTA^Alexa405^ in the UVHis-PAGE approach for detecting His_6_-SUMO in *Escherichia coli* extract. We prepared uninduced and induced samples as a double loading volume (30 µl) and then split them into two independent gels. One copy of the gel was transferred to a PVDF membrane and blocked with BSA for 1 h, immunoblotted for 2 h with anti-His antibody, and imaged with chemiluminescence (Fig. 5*b*). The second copy of the gel was fixed using the fast protocol (12 min). The gel was then stained with Ni^2+^-*tris*NTA^Alexa405^ as described above and imaged both with a regular cellphone camera and with the camera of the FluorChem Q Image analysis system (Fig. 5*c*). After imaging, the same gel was stained with CBB, as described previously (Fig. 5*a*). A clear additional band at ∼15 kDa was visible in the induced sample relative to the uninduced sample, which corresponds to His_6_-SUMO. The presence of the His-tag was directly confirmed by the antibody-based immunoblotting (Fig. 5*b*). The Ni^2+^-*tris*NTA^Alexa405^ signal was highly specific to the confirmed His_6_-SUMO band under both imaging methods. Notably, no additional unspecific bands could be visualized either by the naked eye or by the camera of the analysis system. These experiments clearly illustrate the high specificity of detection of the Ni^2+^-*tris*NTA^Alexa405^ in the UVHis-PAGE approach, even in complex mixture samples.

## Discussion

In this work, we describe UVHis-PAGE, a method for the detection of His-tagged proteins that bypasses the need for antibody-based immunoblotting. This method allows for the visualization of His_6_-tagged protein using a simple UV transilluminator as the excitation source and the naked eye as the detector, down to amounts as low as 5 pmol in SDS-PAGE or 2.5 pmol in blot membrane. This approach uses a Ni^2+^-loaded NTA-based chelator head and a UV-excitable fluorophore with visible emission, which were carefully chosen.

Amine-terminated *mono*NTA (3, 4) and *tris*NTA (5–9) (Fig. 2*a*) represent two popular and commercially available chelator heads that can be directly used for coupling to NHS ester fluorophores. *tris*NTA loaded with Ni^2+^ has been previously optimized to enhance its binding affinity to His_6_-tags by 1000-fold as compared with that of Ni^2+^-*mono*NTA (8). Therefore, our first set of experiments was designed to directly evaluate the performance of these two Ni^2+^-loaded and fluorescently labeled chelator heads in the SDS-PAGE detection of His_6_-tagged proteins. A complete lack of detection was previously shown when Ni^2+^-*tris*NTA was employed in a pre-run staining protocol (17). Ni^2+^-loaded *hexa*NTA is the only known chelator head that can withstand the harsh conditions of the pre-run staining protocol (17). Thus, we switched to a post-run staining protocol (Fig. 1) to directly compare Ni^2+^-*mono*NTA and Ni^2+^-*tris*NTA.

Compared with Ni^2+^-*mono*NTA conjugates, Ni^2+^-*tris*NTA conjugates resulted in a ∼25-fold increase in the detection sensitivity of His_6_-SUMO (Fig. 3). In fact, as per the manufacturer's description, the Atto dyes that were attached to Ni^2+^-*mono*NTA are brighter than their Alexa or cyanine counterparts that were attached to Ni^2+^-*tris*NTA, which makes this result even more remarkable. On a theoretical level, this result also shows that in complex environments, such as in and out of gel diffusion, a several-fold difference in the affinity constant does not necessarily directly translate into the same -fold difference in the detection limit. Nevertheless, given this considerable enhancement, we chose *tris*NTA for the subsequent experiments.

The detection limit of Ni^2+^-*tris*NTA^Alexa647^ of ∼0.1 pmol in post-run staining conditions was similar to that of Ni^2+^-*hexa*NTA^Alexa647^ (∼0.2 pmol) in pre-run staining conditions (17). This similarity opens two potential directions for further investigation. First, this detection limit may be imposed by the conjugated fluorophore itself rather than by the chelator head, a case in which brighter small organic fluorophores should be developed and coupled to these chelator heads to enhance the detection sensitivity. This possibility is also sustained by directly comparing the detection limit of Ni^2+^-*tris*NTA^Alexa647^ (Fig. 3*d*) (∼0.1 pmol) with Ni^2+^-*tris*NTA^Alexa405^ (Fig. 4, *c* and *d*) (∼5 pmol), which, despite using the same chelator head, resulted in a ∼50-fold difference in favor of the brighter Alexa Fluor 647 and the more advanced instrumentation used for its visualization (Fig. 2*c* and Table S1). Alternatively, because Ni^2+^-*hexa*NTA is ∼1000-fold stronger binder than Ni^2+^-*tris*NTA (16) but these two Ni^2+^-loaded MCHs resulted in a similar detection limit (when used under pre-run (for Ni^2+^-*hexa*NTA) and post-run (for Ni^2+^-*tris*NTA) staining conditions, respectively), it is possible that any chelator head may not be used at its maximum capacity under SDS-PAGE pre-run staining conditions. This possibility is also sustained by the completely different behavior of Ni^2+^-*tris*NTA^Alexa647^ under post-run staining conditions in the current study and under the pre-run staining conditions described previously (17).

In addition to the theoretical advantage of directly comparing the staining kinetics using different fluorophore-coupled chelator heads, post-run staining conditions offer several further advantages, as well as a drawback, when compared with pre-run staining conditions. For instance, because *tris*NTA can be employed, post-run staining conditions allow for the efficient detection of the most common form of His-tag, His_6_, in SDS-PAGE. Moreover, because the complex is formed post-run, post-run staining conditions do not induce an undesired upper shift in the apparent molecular weight of the protein of interest. Lastly, the post-run staining conditions protocol allows for gel fixation and later staining without the requirement for immediate imaging. However, this protocol consumes larger amounts of Ni^2+^-*tris*NTA conjugates in the staining step. Nevertheless, both protocols perform similarly well, with a detection limit similar to that of antibody-based chemiluminescent immunoblotting. A comparison of the detection limits and required instrumentation for different His-tag detection assays in PAGE and blotting membranes is summarized in Table S2.

Next, we focused on increasing the ease of detection by changing the fluorophore to Alexa Fluor 405. This dye is UV-excitable and exhibits fluorescence emission in the visible part of the spectrum (Fig. 2*b*). Typical detection usually employs green or red fluorophores, which require specialized excitation sources and detection systems. By coupling Alexa Fluor 405 with Ni^2+^-*tris*NTA, we were able to detect amounts of His_6_-SUMO as low as 5 pmol for SDS-PAGE or 2.5 pmol in blotting membrane using a simple UV transilluminator as the excitation source and the naked eye as the detector. This method of detection showed excellent correlation with the CBB staining method (Fig. 4 and Fig. S3) and was highly specific to the His-tagged protein, even in complex mixture samples (Fig. 5). These results are particularly remarkable in the context of the reduced brightness of UV-excitable dyes such as Alexa Fluor 405, as well as their strong quenching upon conjugation to Ni^2+^-*tris*NTA (Fig. 2*c* and Table S1). Therefore, in the future, such simplified UV excitation–based detection systems with naked-eye visualization could significantly improve with the development of brighter small organic fluorophores that have considerable UV excitation and visible emission.

In conclusion, UVHis-PAGE can be an ideal tool for the rapid and straightforward detection of His-tagged proteins in applications where specialized fluorescence detection is unavailable or traditional antibody-based immunoblotting is too costly or time-consuming. Apart from indicating the presence of a particular epitope, immunoblotting with secondary antibodies, based on both chemiluminescent (28, 29) and fluorescent (28, 30, 31) detection, has been used as a quantitative tool to determine the epitope amount. For a given set of conditions, such as gel percentage and composition or blotting membrane composition, the type and concentration of Ni^2+^-MCH conjugate used, the staining and destaining times, and the imaging parameters, we envision that our methods can also be used as quantitative tools, mainly through the use of a calibration curve similar to the dependence described in Fig. S3. For the methods presented here, all the necessary chemical components are commercially available and, through the use of the well-established amine-NHS chemistry, require uncomplicated experimental conditions for efficient coupling. To clarify the required amount of reagents for implementing our methods, we summarized the starting amount of materials and the final yields, volumes, and concentrations for the three Ni^2+^-loaded *tris*NTA conjugates in Table S3. In a broader sense, the current work also highlights the benefits of using UV-excitable dyes in various assays, which, despite their lower brightness, can offer a simple platform for detection because of the simplified equipment requirements.

## Experimental Procedures

### Protein expression and purification

To express the His_6_-SUMO protein, the empty expression plasmid pE-SUMO (LifeSensors) was transformed into BL21(DE3) *E. coli* expression strain (Novagen). 2 liters of 2× YT (Teknova) media supplemented with 50 mg/liter kanamycin was inoculated from an overnight pre-culture and grown at 37°C. When the cell growth reached an *A*_600_ of 0.8, the expression was induced by the addition of 0.1 mm isopropyl β-d-thiogalactopyranoside (IPTG), and the incubation continued for an additional 4 h at 37°C. From here on, the purification steps were performed at 4°C. The soluble fraction of the cell lysate was applied onto a 5-ml HisTrap HP (GE Healthcare) affinity column, and the protein was eluted with 350 mm imidazole. The eluted protein was concentrated and further purified over a 120-ml Superdex 75 pg size-exclusion column (GE Healthcare). All these steps were performed using an FPLC system. The final purity of the His_6_-SUMO protein was assessed by SDS-PAGE (Fig. S2*a*), followed by quantification using the built-in option of the ImageJ software. The purity was found to be higher than 95%.

### Synthesis of fluorescent multivalent chelator probes

Ni^2+^-*mono*NTA conjugates of Atto550 and Atto647N were purchased from Sigma-Aldrich. *tris*NTA amine was purchased from Toronto Research Chemicals. NHS ester forms of Alexa Fluor 405 and Alexa Fluor 647 were purchased from Thermo Fisher Scientific. NHS ester form of Cy3B was purchased from GE Healthcare. The amine-NHS coupling reactions were performed according to the well-established protocol described in Refs. 8 and 9. The conjugates were purified over a reversed-phase C18 (Sigma-Aldrich) column by using an HPLC system, verified by MALDI-TOF-MS, and loaded with Ni(II), identically to the steps described in Refs. 8 and 9. After incubation with Ni(II), the conjugates were purified over a 1-ml HiTrap Q HP (GE Healthcare) column and eluted with a 0–2.5 m NaCl gradient using an FPLC system. It is worth noting that Ni^2+^-*tris*NTA conjugates of Cy3B and Alexa Fluor 647 were eluted at less than 1 m NaCl concentration, whereas the Ni^2+^-*tris*NTA conjugate of Alexa Fluor 405 required up to ∼1.6 m NaCl for complete elution (Fig. S1). The final yields, volumes, and concentrations for the three Ni^2+^-loaded *tris*NTA conjugates are summarized in Table S3.

### SDS-PAGE running and staining

The target samples of interest were mixed with 5× electrophoresis sample buffer (10% SDS, 500 mm DTT, 50% glycerol, 250 mm Tris-HCl, pH 6.8), heated for 10 min at 95°C, and then loaded onto 10% SDS-PAGE gels (Invitrogen NuPAGE 10% Bis-Tris gels, 10 wells and 1.0 mm thickness). The gels were run in 1× MOPS SDS running buffer (Invitrogen Novex 20× NuPAGE MOPS SDS Running Buffer). The electrophoresis sample buffer intentionally did not contain any loading dye that could interfere with image acquisition.

For CBB staining, the gels were stained with 1× staining solution (40% methanol, 20% glacial acetic acid, 40% water, and 0.3% (w/v) Coomassie Brilliant Blue G-250) while heating in the microwave for 1 min. The gels were then destained with water while heating for 10 min in the microwave.

For Ni^2+^-NTA–based detection, the gels were fixed with 1× fixation solution (40% methanol, 20% glacial acetic acid, and 40% water) by heating in the microwave for 2 min, followed by rinsing with water, and heating again for 10 min in the microwave in water. The gels were then submerged in 1× PBS containing 150 nm Ni^2+^-*mono*NTA^Atto647N^ or Ni^2+^-*tris*NTA^Alexa647^, 300 nm Ni^2+^-*mono*NTA^Atto550^ or Ni^2+^-*tris*NTA^Cy3B^ or 2 μm Ni^2+^-*tris*NTA^Alexa405^. The gels were incubated in these solutions for 1 h with gentle shaking in the dark, rinsed with water, and destained in warm water for varying amounts of time, as indicated in each case.

### Immunoblotting

Following the SDS-PAGE procedure, the proteins were transferred to PVDF membrane with 0.45 μm pore size (Merck) using the sandwich method. The transfer was performed under a constant electric current of 0.39 A for 90 min. The transfer buffer contained 25 mm Tris-base, 192 mm glycine, both dissolved in ddH_2_O, and 20% (v/v) methanol. After the transfer, the membranes were washed once with 1× TBST. The membranes were then blocked for 1 h while shaking at room temperature using 5% (w/v) BSA dissolved in 1× TBST. Following that, the membranes were washed once with 1× TBST. Then, they were incubated with the anti-histidine tag antibody (Bio-Rad, MCA1396) at a concentration of 1 μg/ml for the indicated amount of time, depending on the experiment, as described under “Results.” The proteins were then washed with 1× TBST three times for 5 min each. Next, the membranes were incubated for 30 min at room temperature while shaking with the anti-mouse IgG, HRP-conjugated antibody (Cell Signaling Technology, 7076) at a concentration of 0.1 μg/ml. Finally, the membranes were washed three times for 5 min each and incubated for 2 min with the chemiluminescent substrate (SuperSignal West Pico, Thermo Fisher, 34080) before imaging.

### Gel and membrane imaging

The CBB-stained gels were imaged under white light using an iBright CL1000 system (Thermo Fisher Scientific) or a regular cellphone camera. The antibody-based immunoblotting membranes were imaged with the iBright CL1000 system under the chemiluminescence mode. Ni^2+^-*tris*NTA^Alexa405^–stained gels and membranes were exposed to the UV light generated by the UV transilluminator of the FluorChem Q Image analysis system (Alpha Innotech) and imaged with the camera of the same system or by a regular cellphone camera. The Atto550, Cy3B, Atto647N, and Alexa Fluor 647 conjugate-stained gels were imaged using an Amersham Biosciences Typhoon biomolecular laser scanner (GE Healthcare).

## Data availability

The authors declare that all the data supporting the findings of this study are available within the main text and figures of the manuscript and its supporting information. The source data underlying Fig. 2, *b* and *c* and Fig. S3 are also provided as a source data file that contains the numerical values used for the figure generation.

## Supplementary Material

Supporting Information
