# Supplementary material for: Simplified detection of polyhistidine-tagged proteins in gels and membranes using a UV-excitable dye and a multiple chelator head pair
Source: J Biol Chem. 2020 Jul 9;295(34):12214–23. doi: 10.1074/jbc.RA120.014132 (PMC7443479; doi:10.1074/jbc.RA120.014132)
Supplement: Supporting Information [file supp_295_34_12214__index.html]

Simplified detection of polyhistidine-tagged proteins in gels and membranes using a UV-excitable dye and a multiple chelator head pair — UVHis-PAGE detection of His-tagged proteins — Simplified detection of polyhistidine-tagged proteins in gels and membranes using a UV-excitable dye and a multiple chelator head pair — UVHis-PAGE detection of His-tagged proteins — Supporting Information 

# Simplified detection of polyhistidine-tagged proteins in gels and membranes using a UV-excitable dye and a multiple chelator head pair

## Supporting Information

- Ultraviolet detection of polyhistidine-tagged proteins in polyacrylamide gel electrophoresis and blot membranes using multiple chelator heads - Supporting Figures and their methods
- Supporting Information (to be published online) - Source data file
