# Supplementary material for: Simplified detection of polyhistidine-tagged proteins in gels and membranes using a UV-excitable dye and a multiple chelator head pair
Source: J Biol Chem. 2020 Jul 9;295(34):12214–23. doi: 10.1074/jbc.RA120.014132 (PMC7443479; doi:10.1074/jbc.RA120.014132)
Supplement: Supporting Information [file supp_RA120.014132_160510_2_supp_560961_qd62p1.pdf]

Supporting Information

Simplified detection of polyhistidine-tagged proteins in gels and membranes using a UV-excitable dye and a multiple chelator head pair

Vlad-Stefan Raducanu[#], Ioannis Isaioglou[#], Daniela-Violeta Raducanu[#], Jasmeen S. Merzaban[#], and Samir M. Hamdan[#, *]

[#] King Abdullah University of Science and Technology, Division of Biological and Environmental Sciences and Engineering, Thuwal 23955, Saudi Arabia.

*Corresponding author.
E-mail address: samir.hamdan@kaust.edu.sa (S.M. Hamdan)

Supporting Experimental procedures

Brightness determination for the fluorophore conjugates

For the fluorophores in their free NHS ester form, molecular fluorescence brightness (B^{Free}) was calculated, using reference values for the molar extinction coefficient (ε) at the maximum of absorption (λ_{max}) and for the fluorescence quantum yield (ϕ^{Free}), as previously described in (32):

$$B_{\lambda_{max}}^{Free} = \varepsilon_{\lambda_{max}} \times \phi^{Free} \quad (1).$$

For the Ni^{2+}-*tris*NTA-fluorophore conjugates we opted to determine the fluorescence brightness ($B^{Coupled}$), via the measured fluorescence lifetime (τ), rather than directly through the fluorescence quantum yield which is more error-prone in measurements.

Assuming an energy or electron transfer mechanism in the excited state between the fluorophore and the Ni^{2+} ions loaded on the conjugate (33), implies that the radiative rate of the fluorescence decay (k_r) remains largely unchanged between the free and coupled forms. Only the non-radiative component increases in magnitude to accommodate also the rate of the energy transfer. Under this condition, in both the free and coupled forms, the relationships between the fluorescence quantum yields (ϕ^{Free} and $\phi^{Coupled}$) and measured fluorescence lifetimes (τ^{Free} and $\tau^{Coupled}$) can be written as:

$$\begin{cases} \phi^{Free} = k_r \times \tau^{Free} \\ \phi^{Coupled} = k_r \times \tau^{Coupled} \end{cases} \quad (2).$$

Under the above-mentioned assumption of unchanged radiative rate of the fluorescence decay (k_r), the system of two equalities is algebraically reduced to:

$$\frac{\phi^{Free}}{\phi^{Coupled}} = \frac{\tau^{Free}}{\tau^{Coupled}} \Rightarrow \phi^{Coupled} = \phi^{Free} \times \frac{\tau^{Coupled}}{\tau^{Free}} \quad (3).$$

Adapting the indices in Eq. (1) to the case of the coupled fluorophore and by using Eq. (3), the brightness of Ni^{2+}-*tris*NTA-fluorophore conjugates is immediately given as:

$$B_{\lambda_{max}}^{Coupled} = \varepsilon_{\lambda_{max}} \times \phi^{Coupled} = \varepsilon_{\lambda_{max}} \times \phi^{Free} \times \frac{\tau^{Coupled}}{\tau^{Free}} = B_{\lambda_{max}}^{Free} \times \frac{\tau^{Coupled}}{\tau^{Free}} \quad (4).$$

Here we also assumed that static quenching mechanisms are absent, such that non-fluorescent complexes are not formed and the molar extinction coefficient (ε) remains largely

unchanged. Moreover, we assumed the lack of bathochromic and hypsochromic shifts in the absorption spectra, such that λ_{max} remains largely unchanged. By using reference values for $B_{\lambda_{max}}^{Free}$ and τ^{Free}, with these considerations, the determination of the brightness of the Ni^{2+}-*tris*NTA-conjugated fluorophore is reduced to the measurement of its fluorescence lifetime.

Time-resolved fluorescence lifetime measurements were carried out, as previously described (34,35), using QuantaMaster 800 spectrofluorometer (Photon Technology International Inc.) equipped with a Fianium supercontinuum fiber laser source (Fianium, Southampton, U.K.) operating at 20 MHz repetition rate. Arrival time of each photon was measured with a Becker-Hickl SPC-130 time-correlated single photon counting module (Becker-Hickl GmbH, Berlin, Germany). Measurements were collected under magic angle (54.7°) conditions and photons were counted using time to amplitude converter (TAC). In all measurements, 10,000 counts were acquired. The instrument response function (IRF) was estimated using a Ludox colloidal silica suspension dissolved in water.

Measurements were recorded at room temperature in PBS. All samples were excited at their wavelength of maximum excitation and emission was collected at their wavelength of maximum emission with 5 nm slit width for both excitation and emission. The fluorophore lifetime decays were then obtained using FluoFit software package (PicoQuant) by applying the IRF and fitted to two-exponential decays. The best fit was chosen based on reduced chi-square and randomness of the residuals. The reported lifetimes are the mean of amplitude-averaged lifetimes of three independent replicates.

Analysis of the elution profile of Ni^{2+}-*tris*NTA^{Alexa405} from the HiTrap Q column

The measured A_{400} values of the Ni^{2+}-*tris*NTA^{Alexa405} elution profile from the 1 ml HiTrap Q column were monitored continuously by using the integrated spectrophotometer module of an FPLC system. This measured elution profile was fitted to an Exponentially-Modified Gaussian (exGaussian**,** EMG) profile as previously described in (36-39). First, baseline subtraction routine was applied using the built-in function of the GE Unicorn software. The elution peak was fitted to an EMG profile described by either one of the below equations:

$$\begin{cases} A_{400}(c; h, \mu, \sigma, \tau) = \frac{h\sigma}{\tau}\sqrt{\frac{\pi}{2}}\exp\left(\frac{1}{2}\left(\frac{\sigma}{\tau}\right)^2 - \frac{c-\mu}{\tau}\right)\operatorname{erfc}\left(\frac{1}{\sqrt{2}}\left(\frac{\sigma}{\tau}\right) - \frac{c-\mu}{\sigma}\right) \\ A_{400}(c; h, \mu, \sigma, \tau) = h\exp\left(-\frac{1}{2}\left(\frac{c-\mu}{\sigma}\right)^2\right)\frac{\sigma}{\tau}\sqrt{\frac{\pi}{2}}\operatorname{erfcx}\left(\frac{1}{\sqrt{2}}\left(\frac{\sigma}{\tau} - \frac{c-\mu}{\sigma}\right)\right) \end{cases},$$

where *c* represents the current salt (NaCl) concentration, *h* is the amplitude of the Gaussian which is proportional to the eluted Ni^{2+}-*tris*NTA^{Alexa405} amount and to its molar extinction coefficient, μ and σ are the mean and the standard deviation of the Gaussian part of the model and τ is the relaxation time of the exponential part of the model. Erfc and erfcx are the regular and the scaled

complementary error functions. The elution peak was fitted with one of the above-mentioned functions using the cftool of MATLAB software. After obtaining the fitting parameters μ, σ and τ, the main parameter of interest, namely the mode, i.e. the position of the elution peak maximum was determined as:

$$Mode = \mu - \sigma\sqrt{2}\,\mathrm{erfcxinv}\left(\frac{\tau}{\sigma}\sqrt{\frac{2}{\pi}}\right) + \frac{\sigma^2}{\tau}$$

where erfcxinv is the inverse scaled complementary error function and all the other variables are defined above. Therefore, the parameters μ, σ, τ and the mode have the same units.

Supporting Figures and Tables

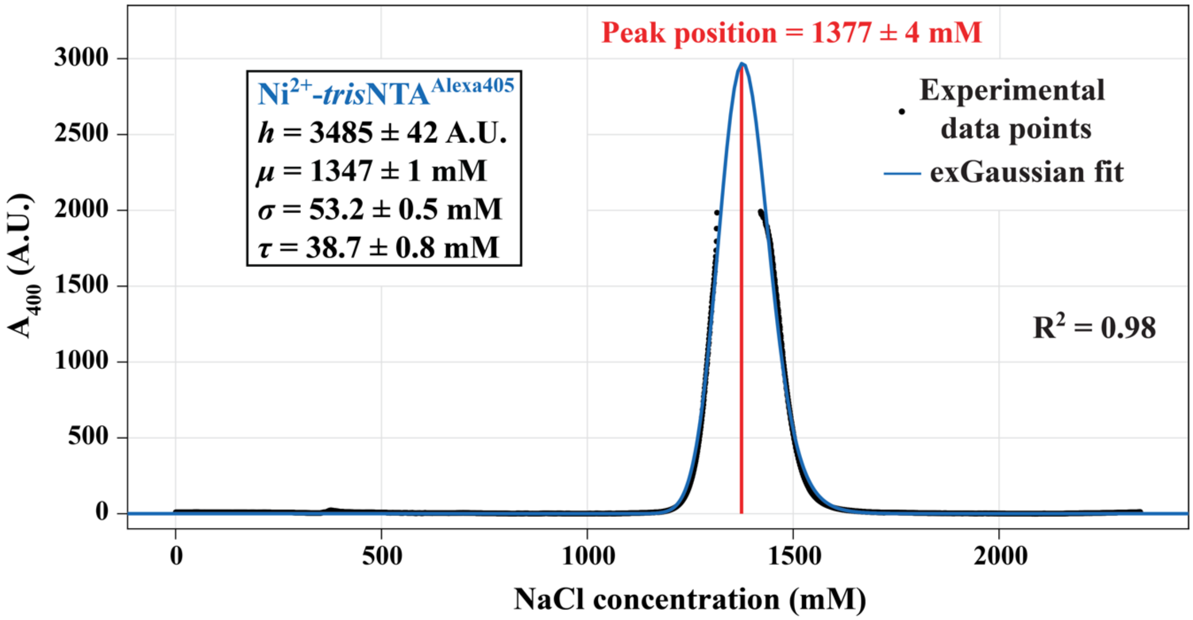

Figure S1. Fitting of the elution peak of Ni^{2+}-*tris*NTA^{Alexa405} from the HiTrap Q 1 ml column by the EMG model. The values of the EMG parameters are shown in the inset table with their 95% confidence interval. The vertical red line represents the position of the maximum of the elution peak as calculated from the fitting parameters as described in the Supporting Experimental procedures section. This value together with its 95% confidence interval is also reported above the peak. Data points above 2000 A.U. were removed, due to spectrophotometer detector saturation.

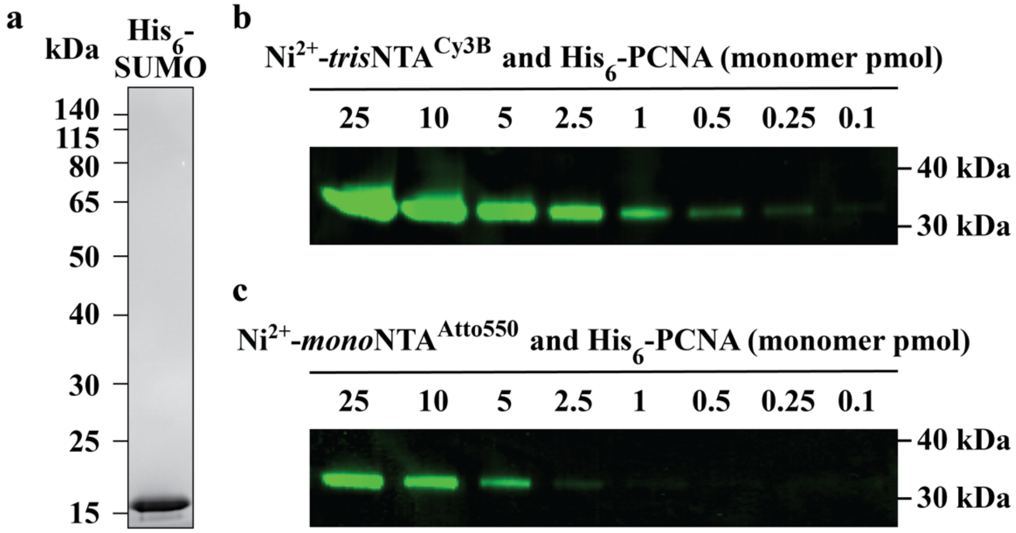

Figure S2. Control SDS-PAGE gels. (a) Image of a CBB-stained SDS-PAGE gel containing the final purified His_6-SUMO protein sample. The molecular weight marker ticks (kDa) correspond to the run of the bands of PageRuler Prestained Protein Ladder (Thermo Fisher Scientific, 26616). Comparison of the detection performance of (b) Ni^{2+}-*tris*NTA^{Cy3B} and (c) Ni^{2+}-*mono*NTA^{Atto550} for His_6-tagged human PCNA (one His_6-tag per PCNA monomer). For both gels the destaining time was 30 min. PCNA amounts are indicated as His_6-tagged PCNA monomer amounts in pmol. Human PCNA tagged at the N-terminus with His_6 was purified identically as described in (40).

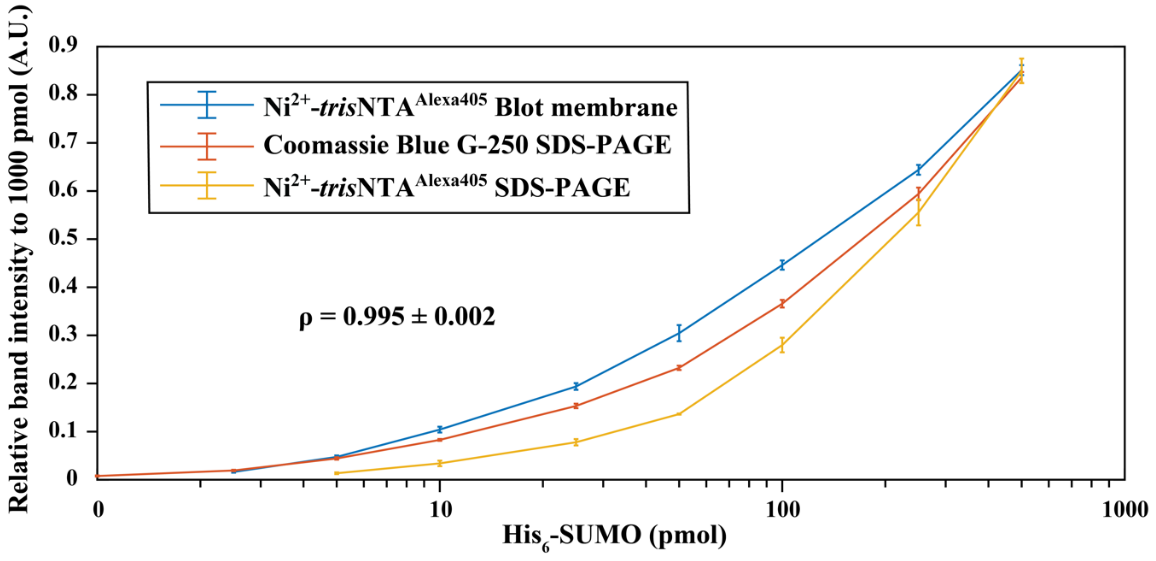

Figure S3. Plot of the normalized band intensities detected by Ni^{2+}-*tris*NTA^{Alexa405}, in both SDS-PAGE and blot membrane, and by Coomassie Blue G-250 (CBB) staining, in SDS-PAGE, from the images presented in Fig. 4 in the main text. The intensity of each band was normalized to the intensity of the 1000 pmol band within the same gel. The correlation coefficient between the Ni^{2+}-*tris*NTA^{Alexa405} and CBB staining in SDS-SPAGE is indicated on the graph together with its 95% confidence interval. Each data point is represented by the average and standard deviation of three independent quantifications using the built-in option of the Image-J software.

Table S1. Photophysical parameters describing the brightness of the Ni^{2+}-*tris*NTA-fluorophore conjugates. All estimated parameters are derived according to the information described in Supporting Experimental procedures section. All values have their source indicated as: [a] as per manufacturer's description, [b] as approximated by the value of the highly similar Cascade Blue dye as per the adjacent reference, [c] as approximated by the value of the highly similar iFluor 405 dye as per manufacturer's description, [d] as described in the adjacent reference and [e] as experimentally determined in the current study.

Fluorophore	Maximum absorption (nm)	Extinction coefficient ($M^{-1}cm^{-1}$)	Free NHS ester form			Ni^{2+}-*tris*NTA-coupled form		Quenching (%)
			Lifetime (ns)	QY	Brightness (A.U.)	Lifetime (ns)	Brightness (A.U.)	
Alexa Fluor 405	401 [a]	35000 [a]	3.8 [b, (41)]	0.91 [c]	31850	1.81 ± 0.04 [e]	15162 ± 340	52.4
Cy3B	559 [a]	130000 [a]	2.4 [d, (34)]	0.67 [d, (42)]	87100	0.87 ± 0.03 [e]	31500 ± 1055	63.8
Alexa Fluor 647	651 [a]	270000 [a]	1.0 [a]	0.33 [a]	89100	0.75 ± 0.01 [e]	66959 ± 780	24.9

Table S2. A summary of the characteristics of various systems that can be used for the detection of His-tagged proteins in PAGE and blot membranes. All the detection limits have their source indicated as: [a] as experimentally determined in the current study, [b] as per manufacturer's description for the Invitrogen Alexa Fluor Plus 800 secondary antibodies and assuming a strong anti-His_6 primary antibody and [c] as described in the adjacent reference. NA denotes not applicable.

Detection system	Detection method	Excitation method	Observation method	Detection limit (pmol)
Coomassie Brilliant Blue G-250	Colorimetric	White light	Naked eye	~1 [a]
Anti-His_6 Ab immunoblotting with HRP secondary Ab	Chemiluminescence	NA	Specialized	~0.1 [a]
Anti-His_6 Ab immunoblotting with fluorescent secondary Ab	VIS-excitation fluorescence	Specialized	Specialized	~0.7 [b]
HisQuick-PAGE (*hexa*NTA, His_{12})	VIS-excitation fluorescence	Specialized	Specialized	~0.2 [c, (43)]
Post-run staining with Ni^{2+}-*tris*NTA-green/red conjugates (His_6)	VIS-excitation fluorescence	Specialized	Specialized	~0.1 [a]
Post-run staining with Ni^{2+}-*mono*NTA-green/red conjugates (His_6)	VIS-excitation fluorescence	Specialized	Specialized	~2.5 [a]
UVHis-PAGE (His_6)	UV-excitation fluorescence	UV transilluminator	Naked eye	~5 [a]
UVHis-Blot (His_6)	UV-excitation fluorescence	UV transilluminator	Naked eye	~2.5 [a]

Table S3. Final yields, concentrations and volumes obtained for the fluorescent Ni^{2+}-*tris*NTA conjugates. All the reagents are described in the Experimental procedures section of the main text.

Fluorophore, NHS ester form	*tris*-NTA starting amount	Dye amount	Dye amount	Conjugate final volume	Conjugate final concentration	Conjugate final yield
Alexa Fluor 405	1 mg ~859.8 nmol	573.2 nmol	~0.59 mg	2000 µl	~78.2 µM	~156.4 nmol
Cy3B			~0.37 mg	1500 µl	~151.2 µM	~226.8 nmol
Alexa Fluor 647			~0.56 mg	1000 µl	~95.1 µM	~95.1 nmol

References

32. Piatkevich, K. D., and Verkhusha, V. V. (2011) Guide to red fluorescent proteins and biosensors for flow cytometry. *Methods Cell Biol* **102**, 431-461
33. Holmes, A. S., Suhling, K., and Birch, D. J. (1993) Fluorescence quenching by metal ions in lipid bilayers. *Biophys Chem* **48**, 193-204
34. Rashid, F., Raducanu, V. S., Zaher, M. S., Tehseen, M., Habuchi, S., and Hamdan, S. M. (2019) Initial state of DNA-Dye complex sets the stage for protein induced fluorescence modulation. *Nat Commun* **10**, 2104
35. Raducanu, V. S., Rashid, F., Zaher, M. S., Li, Y. Y., Merzaban, J. S., and Hamdan, S. M. (2020) A direct fluorescent signal transducer embedded in a DNA aptamer paves the way for versatile metal-ion detection. *Sensor Actuat B-Chem* **304**, 127376
36. Grushka, E. (1972) Characterization of exponentially modified Gaussian peaks in chromatography. *Anal Chem* **44**, 1733-1738
37. Kalambet, Y., Kozmin, Y., Mikhailova, K., Nagaev, I., and Tikhonov, P. (2011) Reconstruction of chromatographic peaks using the exponentially modified Gaussian function. *J Chemometr* **25**, 352-356
38. Raducanu, V. S., Tehseen, M., Shirbini, A., Raducanu, D. V., and Hamdan, S. M. (2020) Two chromatographic schemes for protein purification involving the biotin/avidin interaction under native conditions. *J Chromatogr A* **1621**, 461051
39. Tehseen, M., Raducanu, V. S., Rashid, F., Shirbini, A., Takahashi, M., and Hamdan, S. M. (2019) Proliferating cell nuclear antigen-agarose column: A tag-free and tag-dependent tool for protein purification affinity chromatography. *J Chromatogr A* **1602**, 341-349
40. Lancey, C., Tehseen, M., Raducanu, V. S., Rashid, F., Merino, N., Ragan, T. J., Savva, C. G., Zaher, M. S., Shirbini, A., Blanco, F. J., Hamdan, S. M., and De Biasio, A. (2020) Structure of the processive human Pol delta holoenzyme. *Nat Commun* **11**, 1109
41. Taylor, R. M., Riesselman, M. H., Lord, C. I., Gripentrog, J. M., and Jesaitis, A. J. (2012) Anionic lipid-induced conformational changes in human phagocyte flavocytochrome b precede assembly and activation of the NADPH oxidase complex. *Arch Biochem Biophys* **521**, 24-31
42. Cooper, M., Ebner, A., Briggs, M., Burrows, M., Gardner, N., Richardson, R., and West, R. (2004) Cy3B: improving the performance of cyanine dyes. *Journal of fluorescence* **14**, 145-150
43. Bruchert, S., Joest, E. F., Gatterdam, K., and Tampe, R. (2020) Ultrafast in-gel detection by fluorescent super-chelator probes with HisQuick-PAGE. *Commun Biol* **3**, 138
